# Case Report: Callosal disconnection syndrome manifesting as mixed frontal-callosal-posterior alien hand syndrome following extensive corpus callosum infarct

**DOI:** 10.12688/f1000research.133838.1

**Published:** 2023-05-22

**Authors:** Muhamad Faizal Zainudin, Kar Chuan Soo, Khin Nyein Yin

**Affiliations:** 1 Faculty of Medicine, Universiti Teknologi MARA, Sungai Buloh, Selangor, Malaysia; 2 Department of Rehabilitation Medicine, Hospital Queen Elizabeth, Kota Kinabalu, Sabah, Malaysia; 3 Faculty of Medicine and Health Sciences, Universiti Malaysia Sabah, Kota Kinabalu, Sabah, Malaysia

**Keywords:** alien hand syndrome, corpus callosum, case report, brain infarction, middle cerebral artery stroke, rehabilitation outcome

## Abstract

Alien hand syndrome (AHS) is a rare neurological phenomenon first described by Goldstein over a century ago. The most widely recognized variants in literature are frontal, callosal, and posterior AHS. AHS due to the corpus callosum lesion can occur alone or as part of callosal disconnection syndrome (CDS). This report presents a unique CDS case manifesting clinical features from all three AHS variants, resulting from an extensive corpus callosum infarct.

Our patient exhibited various clinical features from the three AHS variants, which include grasping, groping, and difficulty releasing objects from the hand (anterior); intermanual conflict (callosal); arm levitation, mild hemiparesis, and hemisensory loss (posterior). Additionally, the extensive disruption of the corpus callosal fibers produced neurological manifestations of CDS, such as cognitive impairment, ideomotor and constructional apraxia, behavioral disorder, and transcortical motor aphasia. We employed a range of rehabilitation interventions, such as mirror box therapy, limb restraint strategy, verbal cue training, cognitive behavioral therapy, bimanual hand training, speech and language therapy, and pharmacological treatment with clonazepam. The patient showed almost complete resolution of CDS and AHS features by nine months post-stroke

Our case report highlights distinctive clinical variations of AHS and the challenging correlation between clinical manifestations and neuroanatomical substrates. Future studies are necessary to explore the intricate neural connections and the precise function of the corpus callosum. This can be achieved by combining comprehensive neuropsychological testing with diffusion tensor tractography studies. It is also essential to develop a validated tool to standardize AHS assessment. Finally, the scarcity of evidence in rehabilitation interventions necessitates further studies to address the wide knowledge gap in AHS and CDS management.

## Introduction

The corpus callosum (CC) is the most extensive white matter bundle connecting cortical regions within the two cerebral hemispheres’ frontal, parietal, occipital, and temporal lobes. ^1^ CC fibers contribute to the interhemispheric transfer of cognitive, somatosensory, motor, executive, and visual information. ^2^ Communication disruption between cerebral cortical areas due to CC lesion causes callosal disconnection syndrome (CDS). The corpus callosum receives blood supplies from anterior communicating arteries (ACOM), the pericallosal artery, a branch of the anterior cerebral artery (ACA), and pericallosal branches from the posterior cerebral artery (PCA). ^3^ Given the extensive blood supplies, CC infarcts are a rare occurance. ^3^^,^^4^

The clinical presentation of callosal lesions in each individual is highly variable, depending on location and extent of the CC damage. Even if the lesion location and extent are similar, symptoms can vary widely. ^2^ Patients with a callosal lesion often exhibit severe clinical features ^3^ such as visual agnosia, apraxia, conductive aphasia, alexia, ^5^ tactile and visual anomia, neglect, agraphia, and the dissociative phenomenon commonly known as alien hand syndrome (AHS). ^2^

AHS is defined as involuntary, autonomous, apparently purposeful behaviors in which the affected limb is perceived as being controlled by an external force. ^6^ It was first described by Goldstein ^7^ in 1908 in a stroke patient exhibiting involuntary left-hand movements. Brion & Jedynak ^8^ coined the term ‘alien hand’ in 1972 to describe the experience of the hand as belonging to someone else, which was observed in three patients.

Three principal variants of AHS have been recognized; frontal, callosal, and posterior. The frontal variant typically affects the dominant hand ^9^ and is associated with lesions in the supplementary motor area (SMA), cingulate cortex, dominant medial prefrontal cortex, or the anterior corpus callosum. ^10^ It is characterized by grasping, groping, or compulsive manipulation of tools. The alien hand is perceived as being unpleasant and evokes frustration and anxiety. ^6^ The callosal variant, primarily arising from callosal damage, is characterized by intermanual conflict (IMC) ^10^ of the non-dominant hand, in which one hand acts at cross-purposes with the other, ^6^ likely due to the lack of activation or suppression of inhibitory patterns. ^10^ Together, the frontal and callosal variants make up the anterior AHS. Posterior AHS, on the other hand, is caused by damage to the thalamus, posterolateral parietal, or occipital lobe ^11^ and is characterized by arm levitation, mild hemiparesis, hemispatial neglect, hemisensory loss, and sensory ataxia. ^6^

We describe a case of CDS presenting as mixed AHS, cognitive impairment, ideomotor and constructional apraxia, behavioral disorder, and transcortical motor aphasia. This is the first report of AHS manifesting clinical features from all three variants. Our discussion delves into the distinctive clinical variations, the challenging correlation between clinical manifestations and neuroanatomical substrates, and the implementation of various rehabilitative strategies.

## Case report

This research received ethics approval from the National Medical Research Register Malaysia (NMRR ID-22-02490-XLP).

**Figure 1.**
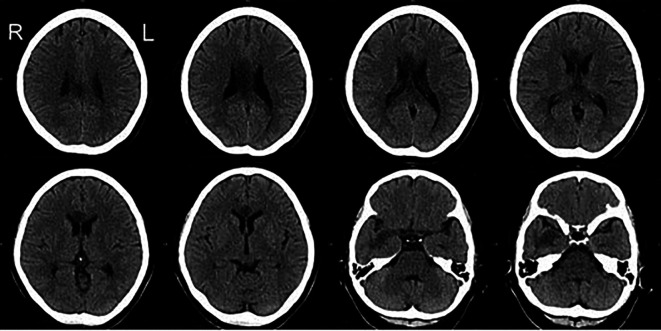
Axial view of the CT Brain. The CT images demonstrate hypodensity over the corpus callosum extending from the right genu to the left side of the splenium and the right temporal lobe. CT, computed tomography.

**Figure 2.**
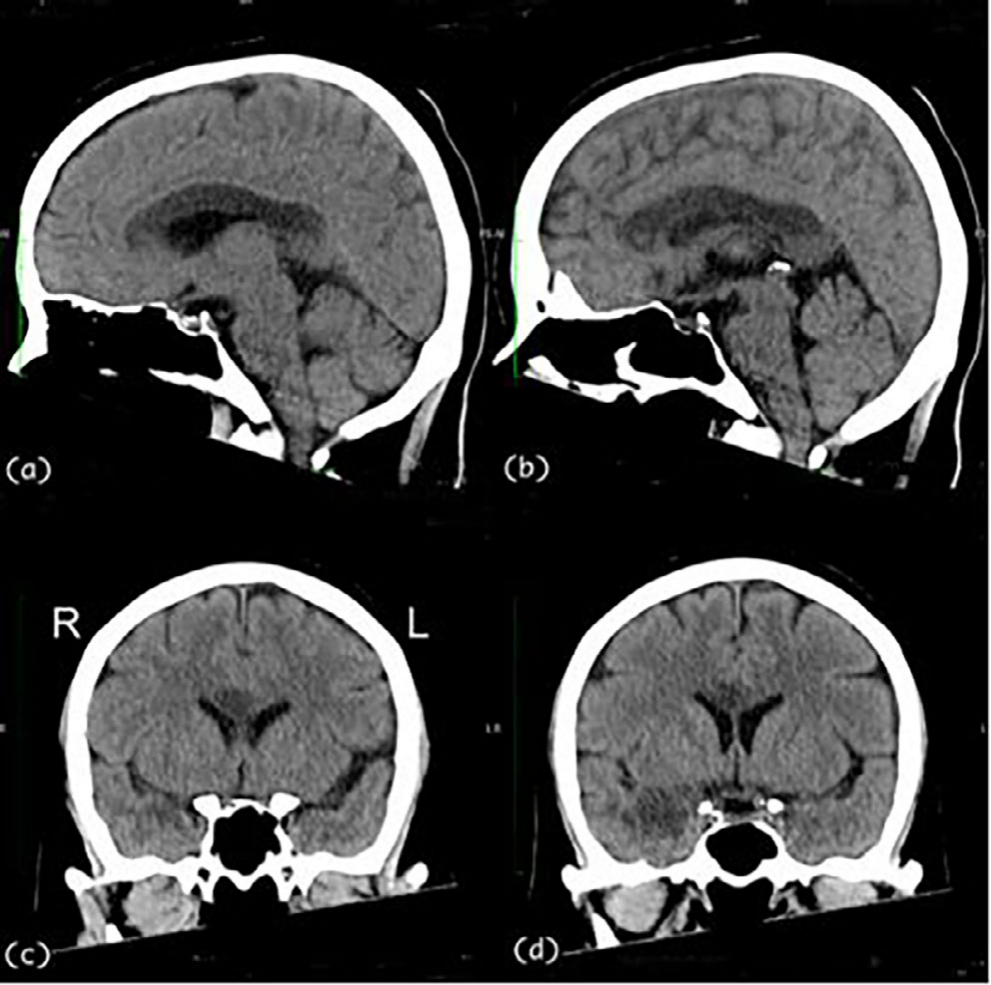
Sagittal [(a) & (b)] and coronal [(c) & (d)] views of the CT Brain. The CT images demonstrate an extensive hypodensity from genu to the splenium of the corpus callosum and the right temporal lobe. CT, computed tomography.

A 57-year-old, right-handed female with risk factors of type II diabetes mellitus and hypertension presented with a sudden onset of left-sided body weakness and slurred speech. The Glasgow Coma Scale (GCS) was 15/15, and her blood pressures were normal. The medical research council (MRC) motor power scale indicated a 4/5 strength in her left upper and lower extremities. Computed tomography (CT) scan of the brain revealed an acute right temporal infarct. The following day, her GCS dropped to 10/15, accompanied by elevated blood pressure. A repeat CT scan displayed an evolution of the right temporal infarct and a new acute corpus callosum infarct extending from the right genu to the left side of the splenium ( Figure 1 and Figure 2). Subsequent CT angiography (CTA) revealed short segment stenosis of the proximal M1 segment of the right middle cerebral artery (MCA), left fetal PCA, and hypoplastic right posterior communicating (PCOM) artery. Three weeks post-stroke, she was transferred to the rehabilitation unit.

### Inpatient rehabilitation

Upon assessment, the patient had global cognitive impairment with a baseline Mini-Mental State Examination (MMSE) score of 14/30. She displayed irritability and disinhibition, often shouting at or striking her caregiver without provocation. Moreover, she was uncooperative when her caregiver attempted to assist her with personal activities of daily living (pADLs), such as getting dressed and bathing. Her mood was labile, with inappropriate crying and laughing episodes, indicative of pseudobulbar affect (PBA). She also had transcortical motor aphasia, evidenced by limited spontaneous speech, difficulty retrieving known words (anomia) and initiating conversation, and long response delay. However, reading and repetition were relatively preserved, and language comprehension was good. Additionally, there was left-sided hemisensory loss but intact proprioception, and there were no signs of hemineglect.

The patient’s mildly paretic left upper extremity manifested abnormal and involuntary movements. It frequently counteracted her right hand’s actions (IMC). For instance, the left hand prevented the right hand from bringing a spoon closer to her mouth while she fed herself. While the right hand attempted to pull up her pants, the left resisted. Additionally, the left hand exhibited independent and purposeless actions. It unintentionally searched for and grasped any objects within sight on the table during meals or therapy sessions (groping & grasping). The patient also struggled to release objects held by her left hand, even when applying force. The left hand failed to follow verbal commands or imitate visual cues (ideomotor apraxia). However, there was no right-left confusion, and the patient remained aware of the unwilled actions performed by her left hand. She expressed anger and frustration by slamming her left hand on the table or using her right hand to strike the left (auto-criticism). Notably, she did not deny ownership of her left hand and considered it part of her body.

The patient received a combination of rehabilitation interventions. During therapy sessions, her affected hand was placed inside a mirror box, preventing her from reaching for objects on the table. The mirror reflected the volitional movement of her right hand, providing the perception that she had control over her left hand’s movement. Additionally, she was taught to keep the left hand in her pocket when it began to move unwillingly (limb restraint). The mirror box and limb restraint strategies were targeted to control the IMC, groping, and grasping. The caregiver received training on verbal cue strategies. She was instructed to verbally prompt the patient to complete her task whenever the IMC occurred. This continued until the patient could complete tasks with her right hand without verbal command. The strategy was also implemented during activities outside the therapy sessions. To alleviate her anger and frustration, the patient underwent cognitive behavioral therapy (CBT). Furthermore, she underwent speech and language therapy, bimanual hand training, pADLs, and ambulation training. Upon discharge (six weeks post-stroke), the patient was moderately dependent in pADLs and could ambulate using a quadripod under her caregiver’s supervision. Her PBA had resolved, and her behavior became more manageable.

### Outpatient rehabilitation

The caregiver reported a new symptom during the first outpatient review two months post-stroke. While walking, the patient’s left hand would spontaneously raise (arm levitation), exhibiting shoulder abduction, elbow flexion, forearm supination, and a fisted hand position. Once elevated mid-air, the arm began shaking uncontrollably. The phenomenon occurred every time she walked, happening more than ten times per day. Groping, grasping, and IMC episodes continued to disrupt her daily activities. However, her speech had improved, with clearer spontaneous speech and shorter response delay. She was more cheerful and displayed appropriate behavior. Although her MMSE score had improved to 27/30, the findings from the visuospatial/executive domain of the Montreal Cognitive Assessment (MoCA) suggested constructional apraxia. To manage the worsening motor symptoms, we prescribed clonazepam 0.5 mg twice daily. The patient continued to receive all previously mentioned interventions as part of her outpatient treatment.

Three months post-stroke, the patient experienced significant improvements. The frequency of arm levitation, groping, and grasping episodes had reduced by 50%, and IMC had entirely resolved. She achieved independence in ambulation without needing a walking aid and could climb stairs while supervised by her caregiver, holding onto the rail for support. The patient could independently perform most pADLs, except for toileting, where she still required some supervision to ensure proper hygiene. Her behavior was appropriate, and the auto-criticism had ceased. To address the remaining arm levitation, groping, and grasping issues, we increased the clonazepam dosage to 1 mg twice daily, aiming for the complete resolution of these motor symptoms.

Six months post-stroke, the patient achieved complete independence in all aspects of pADLs and ambulation. Most CDS and AHS features had resolved, except for arm levitation. She was fully aware of her left arm’s involuntary movement and would place it in her pocket when it began to raise during ambulation. The frequency of groping and grasping episodes had significantly reduced, occurring only once or twice per week, and no longer impairing functionally. Instead of feeling distressed, the patient now found the remaining symptoms amusing. By nine months post-stroke, no further improvements were observed, so the clonazepam treatment was discontinued.

## Discussion

AHS has been a subject of intrigue in the neurological community since its initial description by Goldstein more than a century ago. It remains a relatively uncommon condition. To date, there have been fewer than 200 reported cases, ^10^ with 34 of them representing mixed variant AHS involving a combination of two of the three recognized variants ( Table 1). ^4^^,^^6^^,^^12^^–^^35^ In this report, we discuss an unprecedented case of AHS exhibiting features from all three variants, accompanied by several distinctive clinical and neuroanatomical variations:

(1) The frontal variant symptoms, which were grasping, groping, and difficulty releasing objects from the hand, occurred in the patient’s non-dominant hand, which is an unusual presentation.
(2) The arm levitation, a characteristic of the posterior AHS variant, manifested much later than the other clinical features, suggesting a potential temporal progression in symptom development.
(3) Contrary to the classical description by Brion & Jedynak, our patient did not exhibit denial of ownership of the affected hand, indicating that this symptom may not be a consistent feature across all AHS cases.
(4) Interestingly, the patient’s ACA and ACOM, the primary blood supply to the corpus callosum, were unaffected, as evidenced by the CTA. The observed MCA stenosis could not account for the extensive corpus callosum infarct.
(5) Despite the presence of posterior AHS features, no radiological evidence of thalamic, parietal, or occipital lobe involvement was found.

**Table 1.** Summary of reported cases of mixed variant alien hand syndrome.

Study	Year	Diagnosis	Clinical features		
			Frontal AHS	Callosal AHS	Posterior AHS
Goldstein	1908	Ischemic stroke	Grasping	IMC	-
Goldberg et al.	1981	Ischemic stroke	Grasping, groping	IMC	-
Banks et al.	1989	Ruptured ACOM aneurysm	Groping	IMC	-
Goldberg and Bloom	1990	Ischemic stroke	Grasping	IMC	-
Gottlieb et al.	1992	Ischemic stroke	Groping	IMC	-
Dolado et al.	1995	Ischemic stroke	Groping	-	Arm levitation
Chan et al.	1996	Ischemic stroke	Grasping, groping	IMC	-
Tanaka et al.	1996	Ischemic stroke	Groping	IMC	-
Chan and Ross	1997	Ischemic stroke	Grasping	IMC	-
Chan and Ross	1997	Ischemic stroke	Grasping, groping	IMC	-
MacGowan	1997	Creutzfeldt-Jakob disease	-	IMC	Hemisensory loss
MacGowan	1997	Creutzfeldt-Jakob disease	Grasping	-	Left hemineglect
Fisher	2000	Corticobasal syndrome	Grasping	IMC	-
Fisher	2000	Corticobasal syndrome	-	IMC	Arm levitation
Fisher	2000	Corticobasal syndrome	Grasping	IMC	-
Fisher	2000	Corticobasal syndrome	Grasping	IMC	-
Lavados et al.	2002	Callosal hemorrhage	Grasping, groping	IMC	-
Marey-Lopez et al.	2002	Ischemic stroke	Groping	-	Arm levitation
Pappalardo et al.	2004	Ischemic stroke	Grasping	IMC	-
Giovanetti et al.	2005	Ischemic stroke	Grasping Groping	IMC	-
Kikkert et al.	2006	Ischemic stroke	Grasping	-	Arm levitation left hemineglect
Kikkert et al.	2006	Ischemic stroke	Grasping	IMC	-
Lin et al.	2006	Ischemic stroke	Grasping	IMC	-
Caixeta L	2008	HIV	Grasping	-	-
Haq et al.	2010	Brain herniation	Groping	-	arm levitation
Takenouchi and Solomon	2010	Parry Romberg Syndrome	-	IMC	Arm levitation
Kim et al.	2010	Ischemic stroke	Groping	IMC	-
Pooyania	2011	Ischemic stroke	Grasping	IMC	-
Yuan et al.	2011	Ischemic Stroke	-	IMC	Hemiasthesia
Bartolo	2011	Ischemic stroke	Groping	-	Arm levitation
Rubin et al.	2012	Creutzfeld-Jakob disease	-	IMC	Arm levitation
Nowak et al.	2014	Ischemic stroke	Grasping, groping	IMC	-
Romano et al.	2014	Hemorrhagic stroke	Groping	-	Arm levitation
Qu et al.	2021	Ischemic stroke	Grasping	IMC	-

Abbreviations: ACOM, Anterior Communicating Artery; AHS, alien hand syndrome; IMC, intermanual conflict; HIV, Human Immunodeficiency Virus.

The complex and diverse clinical manifestations of AHS have made anatomical localization challenging. Past studies have highlighted similar discrepancies between clinical presentation and radiological evidence. For instance, Pappalardo et al. ^26^ reported a case of parieto-occipital infarct that exhibited compulsive manipulation, difficulty in releasing tools, and IMC without any posterior AHS features. Similarly, Rafiei et al. ^36^ reported a case of posterior AHS following a left pontine hemorrhage. Although different subtypes of AHS have been distinguished, its clinical diversity has resulted in inconsistent and disputable classification systems. ^4^ While the three-variant classification is commonly used, alternative classification systems have been proposed. Aboitiz et al., ^37^ for example, put forth a five-category classification system that includes: (i) diagonistic dyspraxia and related syndromes, (ii) alien hand, (iii) way-ward hand and related syndromes, (iv) supernumerary hands and (v) agonistic dyspraxia. Additionally, some researchers have suggested classifying AHS into motor (anterior AHS) and sensory (posterior AHS) variants. ^4^

Scepkowski and Cronin-Golomb ^38^ underlined three factors that contribute to the difficulty in systematically studying the AHS:

(1) Its rarity relative to other neurological disorders, forcing reliance on case reviews. This limits the ability to examine a large, diverse population of AHS patients, making it difficult to generalize findings and draw definitive conclusions.
(2) Its transient nature, in most cases, poses challenges for long-term study, complicating the process of capturing and measuring the full range of clinical manifestations.
(3) The coincidental occurrence of other behavioral dysfunctions, such as hemiparesis, motor neglect, or neglect of visual hemispace, hinders the assessment process.

These factors, combined with the lack of uniformity in assessment methods, including both behavioral and neuroimaging tests, impede the establishment of clear subtypes of alien-hand phenomena.

Different pathophysiological mechanisms likely contribute to the diverse behaviors observed in AHS. ^4^ Elucidating them requires a deep understanding of the functional anatomy of the highly intricate callosal network. Conventional radiological modalities such as CT and magnetic resonance imaging (MRI) do not have the capacity to visualize the interhemispheric CC fiber connections. ^2^ The emergence of Diffusion Tensor Tractography (DTT) study in recent years has enabled a 3-dimensional visualization of the CC architecture and integrity, providing a more in-depth analysis into the clinical-anatomical correlation of AHS. ^2^^,^^39^

Isolated and extensive CC infarcts are rare, with only a few reported cases. In 1999, Lausberg et al. ^40^ reported a case of CDS following an infarct of the total length of the corpus callosum, manifesting callosal AHS. Using comprehensive neuropsychological investigations, the authors demonstrated that despite left-handedness, the CDS is analogous to right-handers revealing left-hemispheric dominance for language and praxis. In 2011, Yuan et al. ^4^ described a case of mixed callosal-posterior AHS following an extensive corpus callosum infarct involving the genu, body, and splenium. The authors combined diffusion-weighted imaging (DWI) with magnetic resonance angiography (MRA) to determine the anatomical substrate underpinning the clinical presentation. In 2013, Jang et al. ^2^ reported a case of extensive corpus callosum infarct manifesting CDS and frontal-posterior AHS. In addition to neuropsychological testing, a DTT study was performed, followed by a comparison with the DTT findings of three normal subjects. The DTT revealed disruption of most interhemispheric CC fibers, except for those in the anterior genu and posterior splenium, congruent with the patient’s neurological manifestation. In this report, the lesion was almost exclusively confined to the corpus callosum, sparing the frontal, parietal, and occipital lobes, and the thalamus. The unique neurological manifestations, therefore, can be attributed to the callosal network disruption, causing bihemispheric disinhibition or interhemispheric disconnection. ^10^ The variations in AHS manifestation in these four reports solidify the hypothesis that even if the corpus callosum lesions are similar in location and extent, the symptom manifestations can still vary. ^2^ This report is limited by the lack of neuropsychological and radiological evidence due to service unavailability. However, we believe that if given access to these resources, combining neuropsychological assessments with DTT investigations could significantly contribute to uncovering the underlying pathomechanism of AHS in our patient.

AHS may hinder rehabilitation and can easily be overlooked or misinterpreted. ^31^ Recognizing AHS is vital because its different manifestations may require different therapeutic approaches. ^6^ Due to its rarity, evidence-based rehabilitation interventions for AHS are scarce. To date, there are no approved or recommended therapies for AHS. Management strategies are based on anecdotal reports of both pharmacological and behavioral interventions. For the anterior variant, suggested interventions include sensory tricks, distracting tasks, CBT, and verbal cue. On the other hand, the posterior variant may benefit from treatments such as clonazepam, botulinum toxin, visualization strategies, and spatial recognition tasks. ^10^

In mirror box therapy, the congruency between motor intention and visual feedback elicited by the vision of the intact hand moving through the mirror improves the voluntary control of the alien hand. ^35^ However, due to reliance on sensorimotor integration, this approach may only work for posterior AHS, as anterior AHS seems to depend on a failure to inhibit motor behavior of the non-dominant hemisphere. ^41^ Clonazepam possibly potentiates thalamic GABAergic circuitry, resulting in either reducing the arm’s oversensitivity to external stimuli or dampening the internal stimulus driving the AHS. ^14^ The limb restraint strategy likely provided enough stimulation for the sensory spinal grasp reflex to achieve accommodation, thus inhibiting the movement. ^10^ Interestingly, in the case presented in this report, the IMC showed the best response to these interventions when compared to the other motor symptoms.

Educating the patient and caregiver is essential to managing AHS, as it can help them cope with the challenging behavior associated with this condition. ^31^ Caregiver involvement plays a significant role in optimizing the rehabilitation outcome. For instance, the verbal cue intervention necessitates a strong commitment from the caregiver to deliver the strategy consistently to achieve positive results. Addressing anxiety and fear is also crucial and may involve using anxiolytics and appropriate behavioral therapies guided by experienced psychiatrists and psychologists. ^10^ Unfortunately, none of the discussed interventions have demonstrated long-term benefits. Ergo, impairment-focused and multidisciplinary interventions may be the most appropriate for managing AHS.

Our limited understanding of the AHS pathophysiology has complicated the accurate prediction of its natural history. ^10^ Previous studies have reported mixed prognoses. In some cases, there was a complete resolution of symptoms. ^4^^,^^13^^,^^26^^,^^31^ However, other reports documented the persistence of symptoms, despite some improvement. ^6^^,^^24^^,^^31^ In the case presented here, although some motor symptoms persisted, they were neither disturbing nor impairing functionally.

In conclusion, mixed variant AHS is an exceptionally rare and intriguing neurological phenomenon. To our knowledge, this is the first report of an extensive corpus callosum infarct displaying features from all three recognized AHS variants as part of the CDS. This case underscores the unique and distinctive clinical variations and showcases a challenging correlation between clinical manifestations and neuroanatomical substrates. Despite being recognized as a clinical entity for over a century, establishing a clear correlation between anatomical and clinical findings in AHS remains elusive, ^10^ and current theories on its underlying mechanisms are largely speculative. ^6^ Future research is essential to unravel the intricate neural connections and the precise function of the corpus callosum. This can be best achieved by combining comprehensive neuropsychological testing with DTT studies. Refining AHS classification systems will be crucial as research continues to uncover the underlying pathomechanism and anatomical correlates. Additionally, there is a need for the development of a validated tool to standardize the AHS assessment, and the limited evidence available on rehabilitation interventions warrants further exploration. By fostering continued investigation and collaboration, we can deepen our understanding of AHS and enhance therapeutic options for those affected by this rare and enigmatic neurological disorder.

## Consent

The authors sought written informed consent prior to the submission of this case report from the patient and her primary caregiver, her eldest daughter. We sought permission to share any clinical photo, video, laboratory investigation result, and imaging study from them before submitting the report.

## Data Availability

No data are associated with this article.
