# Supplementary material for: Case Report: Callosal disconnection syndrome manifesting as mixed frontal-callosal-posterior alien hand syndrome following extensive corpus callosum infarct
Source: F1000Res. 2024 Jun 17;12:529. Originally published 2023 May 22. [Version 2] doi: 10.12688/f1000research.133838.2 (PMC11074696; doi:10.12688/f1000research.133838.2)
Supplement: Peer review history

# Reviewer report: Reviewer response for version 1

**DOI:** 10.5256/f1000research.146853.r258091

**Published:** 2024-05-06

**Authors:** Victor W Mark

**Affiliations:** 1 University of Alabama at Birmingham, Birmingham, USA

The authors contribute a unique observation, which is the combination of all three kinds of alien hand that are generally recognized. The patient's background, clinical findings, clinical course, and supporting brain imaging are included. 

 The authors in addition have completed a thorough review of the pertinent literature that pertains to alien hand syndrome. Their Table of the literature is very helpful to readers interested in alien hand sign. 

 Some suggested revisions:

 1) The abstract and Introduction attribute the first report of alien hand to Goldstein 1908. Most review papers point to this paper.

 However, in my own literature review, I find alien hand in yet an earlier report, from 1907: Van Vleuten CF. Linksseitige motorische Apraxie. Ein Beitrag zur Physiologie des Balkens. Z Psychiatr 1907;64:203-239. The patient that was described succumbed to a brain tumor, preceded by involuntary movements, including strong involuntary grasp reflex, left hand not participating with undressing even though the patient understood the command, not stretch out the left forefinger on command, complains he cannot understand why the difficulty. The problem is inconsistent. At other times the left hand moves to part of the surroundings in contrast to where the patient was instructed, unlike the right hand. When handed a pencil (with what hand not stated) and asked to write instead he brings it to his mouth 10 times, despite his saying "I do not want to moisten the pencil." And so forth. 

 This report has only rarely been mentioned in neuroscience literature, and not with attention to alien hand except in a superficial manner (cf Graff-Radford NR,eta l, 1987;[Ref 1]

 2) Under Patient rehabilitation, the passage that the patient had a "left-sided hemisensory loss," but without details. Unclear whether this reflected light touch, pin, or otherwise. 

 3) Under Outpatient rehabilitation, the top paragraph indicates that the patient's left hand would rise (levitation) that occurred specifically during walking. While this does indeed suggest the posterior form of alien hand, this also suggests a form of paroxysmal kinesigenic dyskinesia. These may not be mutually exclusive diagnoses; I suggest review this possible alternate diagnosis.

Are enough details provided of any physical examination and diagnostic tests, treatment given and outcomes?

Yes

Is the case presented with sufficient detail to be useful for other practitioners?

Yes

Is sufficient discussion included of the importance of the findings and their relevance to future understanding of disease processes, diagnosis or treatment?

Yes

Is the background of the case’s history and progression described in sufficient detail?

Yes

Reviewer Expertise:

Multiple sclerosis rehabilitation, functional neurological disorder study, case reports of alien hand syndrome.

I confirm that I have read this submission and believe that I have an appropriate level of expertise to confirm that it is of an acceptable scientific standard.

## References

[1] : Callosal apraxia. *Neurology* .1987;37(1) : 10.1212/wnl.37.1.100 100-5 10.1212/wnl.37.1.100 3796825

# Response

**Published:** 2024-06-11

**Authors:** Khin Nyein Yin

**Affiliations:** Universiti Malaysia Sabah, Malaysia

We thank you and appreciate your review. The feedback you provided has helped improve the quality and clarity of this manuscript. Below are our responses to your inquiries.

 1.    The Abstract and Introduction attribute the first report of alien hand to Goldstein 1908. Most review papers point to this paper. However, in my own literature review, I find alien hand in yet an earlier report, from 1907 (Van Vleuten CF). This report has only rarely been mentioned in neuroscience literature, and not with attention to alien hand except in a superficial manner (cf Graff-Radford NR).

 Response

 Thank you for this valuable information that we missed. Information on the Van Vleuten paper is scarce, and most papers only briefly reference the article, including the reference you suggested. We have amended the information on the earliest reported cases of AHS by referencing Van Vleuten’s study. The report by Van Vleuten can be found in the following sentences:

 “…..first described by Van Vleuten……” (Abstract)

 “It was first described by Van Vleuten in 1907 in a patient with a left-hemisphere brain tumor that had invaded the corpus callosum and exhibited right-hand grasping.” (Page 3, lines 18-20)

 “AHS has been a subject of intrigue in the neurological community since its initial description by Van Vleuten  more than a century ago.” (Page 6, lines 138-139)

 2.    Under Patient Rehabilitation, the passage that the patient had a “left-sided hemisensory loss,” but without details. Unclear whether this reflected light touch, pin, or otherwise.

 Response

 We have added the information to the revised manuscript:

 “Additionally, there was left-sided hemisensory loss in both pin-prick and light touch sensation but intact proprioception,…..” (Page 5, lines 76-78)

 3.    Under Outpatient rehabilitation, the top paragraph indicates that the patient’s left hand would rise (levitation) that occurred specifically during walking. While this does indeed suggest the posterior form of alien hand, this also suggests a form of paroxysmal kinesigenic dyskinesia. These may not be mutually exclusive diagnoses; I suggest review this possible alternate diagnosis.

 Response

 Thank you for the suggestion. We have expanded the discussion on the arm levitation and the consideration of PKD as a differential diagnosis:

 “The arm levitation presentation in our patient could also indicate paroxysmal kinesigenic dyskinesia (PKD), a rare disorder characterized by involuntary movements such as dystonia and choreoathetosis, precipitated by sudden movement or startle (new reference no. 2). However, several characteristics suggest that PKD is less likely than posterior AHS. Firstly, PKD attacks are typically triggered by abrupt movement, an increase in speed, amplitude, or strength, the sudden addition of new actions during ongoing steady movements, or a change in direction (new reference no. 3). In our case, the arm levitation was triggered only by walking—a step-wise, voluntary movement that did not involve sudden movement. The speed and direction of walking did not affect the manifestation of arm levitation. Secondly, the duration of PKD attacks is typically brief, mainly lasting less than a minute (new reference no. 3). In contrast, the arm levitation in our patient persisted throughout her ambulation. Thirdly, most cases of PKD are preceded by a premonition or aura (new reference no. 2), which was absent in our patient. Lastly, new PKD attacks cannot be induced for a refractory period of approximately 20 minutes (new reference no. 3). However, arm levitation could easily manifest upon walking without any such refractory period in our patient. Therefore, the arm levitation in this patient aligns more with the posterior AHS presentation than with PKD.” (Page 8-9, lines 161-177)

# Reviewer report: Reviewer response for version 1

**DOI:** 10.5256/f1000research.146853.r266220

**Published:** 2024-05-03

**Authors:** Stefano L Sensi, Matteo Alessandro De Rosa

**Affiliations:** 1 University G. d'Annunzio of Chieti-Pescara, Chieti, Italy; 2 Department of Neuroscience, Imaging and Clinical Sciences, G. d’Annunzio, University of Chieti-Pescara, Chieti, Chieti, Italy

- It would be important to understand the timing of the second TC scan and why an MRI was not performed. An MRI would certainly have given further information about the features and anatomical sites involved.
- Has a neuropsychological evaluation been performed to assess the cognitive domains involved? It could have been very useful for an assessment at baseline and to evaluate improvements.
- Finally, how do you explain the occurrence of AL so far from the acute event?, Please explain these aspects in more detail.
- Otherwise, the article is very interesting, informative  and well-written.

Are enough details provided of any physical examination and diagnostic tests, treatment given and outcomes?

Partly

Is the case presented with sufficient detail to be useful for other practitioners?

Yes

Is sufficient discussion included of the importance of the findings and their relevance to future understanding of disease processes, diagnosis or treatment?

Yes

Is the background of the case’s history and progression described in sufficient detail?

Partly

Reviewer Expertise:

NA

We confirm that we have read this submission and believe that we have an appropriate level of expertise to confirm that it is of an acceptable scientific standard.

# Response

**Published:** 2024-06-11

**Authors:** Khin Nyein Yin

**Affiliations:** Universiti Malaysia Sabah, Malaysia

Thank you for your positive review. We appreciate the comments and have provided responses to your inquiries below:

 1.    It would be important to understand the timing of the second CT scan and why an MRI was not performed. An MRI would certainly have given further information about the features and anatomical sites involved.

 Response:

 The second CT was done on the day the patient had a drop in her GCS. We have added this information to the text:

 “A repeat CT scan was done urgently on the same day and displayed….” (Page 4, line 50)

 An MRI was not performed due to service limitations. We expanded the study limitations in a separate paragraph:

 “In our setting, a tertiary government hospital with heavy patient loads and limited resources, there is no in-house clinical psychologist. Additionally, MRI availability is restricted and reserved for a limited number of cases. Given that an MRI would not alter the management of the stroke itself, the request for an MRI in this case was not granted. The patient was advised to consider obtaining an MRI and neuropsychological evaluation at a private center; however, these investigations were not performed due to financial constraints.” (Page 10, lines 233-239)

 2.    Has a neuropsychological evaluation been performed to assess the cognitive domains involved? It could have been very useful for an assessment at baseline and to evaluate improvements

 Response

 A neuropsychological evaluation would have been invaluable to the case. Unfortunately, we do not have an in-house clinical psychologist. This is explained in the study limitations paragraph:

 “In our setting, a tertiary government hospital with heavy patient loads and limited resources, there is no in-house clinical psychologist. Additionally, MRI availability is restricted and reserved for a limited number of cases. Given that an MRI would not alter the management of the stroke itself, the request for an MRI in this case was not granted. The patient was advised to consider obtaining an MRI and neuropsychological evaluation at a private center; however, these investigations were not performed due to financial constraints.” (Page 10, lines 233-239)

 3.    Finally, how do you explain the occurrence of AL so far from the acute event? Please explain these aspects in more details.

 Response

 The patient was treated in a different hospital during the acute event. The nearly three-week stay was complicated by medical issues such as upper gastrointestinal bleeding and anemia requiring blood transfusions, and poorly-controlled diabetes. During medical stabilization, there was no dedicated caregiver accompanying her in the ward. She was reported to be sleeping in bed most of the time, and there was no documentation of abnormal movement from the referring hospital. Without eyewitness or documented evidence, it is difficult to ascertain the onset of AHS itself.
